# cAMP Modulators before In Vitro Maturation Decrease DNA Damage and Boost Developmental Potential of Sheep Oocytes

**DOI:** 10.3390/ani11092512

**Published:** 2021-08-26

**Authors:** Daniela-Alejandra Medina-Chávez, Irene Sánchez-Ajofrín, Patricia Peris-Frau, Carolina Maside, Vidal Montoro, Rocío Fernández-Santos, José Julián Garde, Ana Josefa Soler

**Affiliations:** SaBio IREC (CSIC-UCLM-JCCM), ETSIAM, Campus Universitario s/n, 02071 Albacete, Spain; daniela.medina@uclm.es (D.-A.M.-C.); irene.ssanchez@uclm.es (I.S.-A.); patricia.peris@uclm.es (P.P.-F.); carolina.maside@uclm.es (C.M.); vidal.montoro@uclm.es (V.M.); mrocio.fernandez@uclm.es (R.F.-S.); julian.garde@uclm.es (J.J.G.)

**Keywords:** sheep, embryo, oocyte, in vitro maturation, DNA damage, cAMP, IBMX, forskolin

## Abstract

**Simple Summary:**

Oocyte in vitro maturation has massive potential for the generation of great numbers of embryos for research and for the application of assisted reproductive technologies, such as in vitro embryo production. However, the developmental ability of in vitro matured oocytes is lower than those matured in vivo. Here, incubating the oocytes with cAMP modulating agents for two hours before in vitro maturation decreased oocyte DNA damage and increased the number of embryos generated after in vitro fertilization. The present findings could help to develop new methods to improve the quality and developmental potential of in vitro matured oocytes.

**Abstract:**

To date, the underlying mechanisms by which cAMP modulators act during in vitro maturation to improve oocyte developmental competence are poorly understood. Here, we sought to fill this knowledge gap by evaluating the use of phosphodiesterase inhibitor 3-isobutyl-1-methylxanthine (IBMX) and adenylyl cyclase activator forskolin during a culture period of 2 h before in vitro maturation (pre-IVM) on the nuclear and cytoplasmic maturation features in essential organelles, cumulus cells activity, and in vitro developmental potential of sheep oocytes. Results showed that pre-IVM treatment significantly decreased (*p* < 0.05) the DNA damage of mature oocytes (pre-IVM = 2.08% ± 3.51% vs. control = 20.58% ± 3.51%) and increased (*p* ≤ 0.05) expanded blastocyst rates compared to the control (from the total of oocytes: pre-IVM = 23.89% ± 1.47% vs. control = 18.22% ± 1.47%, and from the cleaved embryos: pre-IVM = 45.16% ± 1.73% vs. control = 32.88% ± 1.73%). Considering that oocytes are highly vulnerable to the accumulation of DNA damage because of exposure to in vitro culture conditions, our results suggest that the modulation of intra-oocyte cAMP levels with forskolin and IBMX before IVM might afford oocytes a more effective DNA repair mechanism to overcome damage obstacles and ultimately improve developmental competence. This previously unappreciated action of cAMP modulators could help to develop improved methods for assisted reproduction technologies in animal and clinical research.

## 1. Introduction

Despite being one of the most promising assisted reproductive technologies, in vitro maturation (IVM) has become a major limiting factor for the in vitro production (IVP) of viable embryos in different species [1]. Even with the many advances, the developmental potential of IVM oocytes is significantly lower than that of their in vivo matured counterparts, which highlights the importance of optimizing IVM protocols [2]. During maturation, oocytes acquire their developmental competence, i.e., the ability to be fertilized and complete pre-implantation embryo development, through the modulation and coordination of both nuclear and cytoplasmic events [3]. Mimicking these processes in vitro is challenging and might be one of the leading causes of impaired IVM.

Before ovulation, oocytes arrest at the prophase I of the meiotic cycle [4]. During this period, mural granulosa cells produce meiosis-inhibiting factors that stimulate the production of cyclic guanosine 3′,5′-monophosphate (cGMP) in cumulus cells [5,6]. The cGMP produced diffuses into the oocyte through gap junctions and inhibits the activity of phosphodiesterase 3A (PDE3A) [7], an oocyte-specific phosphodiesterase that degrades cyclic adenosine monophosphate (cAMP) [8]. This intricate pathway maintains high basal cAMP levels, thereby sustaining meiotic arrest [9]. After the luteinizing hormone (LH) surge, disruption of gap junctional communications between the oocyte and cumulus/granulosa cells is followed by a decrease in cGMP levels, allowing PDE3A to selectively hydrolyze cAMP molecules in the oocyte and induce meiotic resumption [10]. Simultaneously, a series of complex programmed events, including the redistribution of cytoplasmic organelles, transcription of mRNAs, and the modification of proteins, occur in oocytes to complete cytoplasm maturation [11].

Oocytes can also undergo nuclear and cytoplasmic maturation in vitro. Still, the two processes are greatly unsynchronized since the physical removal of cumulus-oocyte complexes (COCs) from antral follicles spontaneously resumes meiosis [12]. This uncontrolled event may also cause partial or incomplete cytoplasmic maturity and eventually compromise the resulting embryos’ developmental potential [13].

For this reason, alternative IVM systems that temporarily maintain high intra-oocyte concentrations of cAMP, and thus meiotic arrest, have been proposed (reviewed in [14,15]). These systems comprise two steps: a pre-IVM stage that blocks the spontaneous resumption of meiosis and prolongs oocyte-cumulus cells’ gap junction communications, and an IVM stage to induce meiotic resumption and oocyte maturation [16,17,18,19,20,21,22]. However, the results obtained so far are still contradictory due to the variability between species and the low robustness of these methods since subtle differences in laboratories’ methodology may lead to different outcomes [23]. Furthermore, little information is available about the impact of such treatments on the oocyte at a molecular level, particularly concerning cytoplasm maturation.

Hence, the aim was to evaluate cAMP modulators forskolin and IBMX effects on completion of meiosis, cytoplasmic reorganization, and cumulus cells’ activity in sheep COCs, and their contribution to subsequent embryonic development. In this context, the present study examined oocyte and cumulus cell quality parameters that have not been investigated before, especially in the ovine species.

## 2. Materials and Methods

All chemicals and reagents were purchased from Merck Life Sciences (Madrid, Spain) unless otherwise indicated.

### 2.1. Oocyte Collection and Pre-Maturation Culture

Ovaries from Manchega or mixed-breed adult ewes of around 6 years old (approximately 450; 90 in each of the 5 replicates) were collected at a slaughterhouse and transported in saline solution (8.9 g/L NaCl #S9625), supplemented with penicillin (0.1 g/L #P3032) and streptomycin (0.1 g/L #S6501) at 25 °C for approximately 2 h. Immature cumulus-oocyte complexes (COCs) were retrieved from the follicles using a scalpel blade in 2 mL of collection medium (TCM199 medium (#M4530), supplemented with 4 µL/mL of gentamicin (#G1272), 2.38 mg/mL of HEPES (#H4034), 2 µL/mL of heparin (Hospira, Lake Forest, IL, USA; #654753.3), and 4 µL/mL of gentamicin (#G1272)). Immediately, the COCs with clear or moderately granular ooplasm surrounded by at least three layers of packed cumulus cells were selected and homogeneously distributed in two types of selection media: control (TCM199 medium, supplemented with 2.38 mg/mL of HEPES and 4 µL/mL of gentamicin) and pre-IVM (TCM199 medium, supplemented with 2.38 mg/mL of HEPES, 4 µL/mL of gentamicin, 500 µM of 3-isobutyl-1-methylxanthine (IBMX, Thermo Fisher Scientific, Barcelona, Spain; #PHZ1124), and 100 µM of forskolin (Cayman Chemicals, Ann Arbor, MI, USA; #HY-15371)). Then, the COCs were washed in TCM199 and 4 µL/mL of gentamicin (control) or TCM199, 4 µL/mL of gentamicin, 500 µM of IBMX, and 100 µM of forskolin (pre-IVM). Subsequently, control COCs were subjected to IVM, while pre-IVM COCs were incubated in TCM199, 4 µL/mL of gentamicin, 500 µM of IBMX, and 100 µM of forskolin for 2 h at 38.5 °C, in 5% CO_2_ and maximal humidity before IVM. The rationale behind supplementing the selection and wash media with cAMP modulators before pre-IVM culture was to avoid cAMP levels dropping immediately after COCs’ collection [21].

### 2.2. Measurement of cAMP Levels in Denuded Oocytes

Intra-oocyte cAMP concentration was assessed in pools of 10 oocytes (*n* = 100, 5 replicates) by the chemiluminescent immunoassay (CLIA) using the DetectX© High-Sensitivity Direct Cyclic AMP Kit, Acetylated Format (Arbor Assays, Ann Arbor, MI, USA; #K019-C1). Briefly, after a 2 h incubation with cAMP modulators, some COCs were denuded by pipetting in 500 µL of PBS-PVA and 500 µM of IBMX, and washed to remove debris from the cumulus cells. Denuded oocytes were transferred to a 1.5 mL tube containing 125 µL of Sample Diluent, immediately frozen in liquid nitrogen, and stored at –80 °C until the analysis day. The CLIA assay was performed according to the manufacturers’ acetylated protocol. All reactions were read on a Sinergy HT luminescence plate reader (BioTek-Agilent Technologies, Santa Clara, CA, USA) at 0.1 s reading time per well. The cAMP concentration was calculated by interpolating luminescence units on a standard curve generated from known amounts of cAMP with the MyAssays application.

### 2.3. In Vitro Maturation

The selected COCs from control and pre-IVM were homogeneously distributed in 4-well plates with 500 µL of maturation medium: TCM199 and 4 µL/mL of gentamicin, 100 µM of cysteamine (#M6500), 10 µg/mL of follicle-stimulating hormone (FSH), and 10% fetal calf serum (#F0804). Then, medium was covered in mineral oil (Nidacon, Gothenburg, Sweden; #NO-400K), and COCs were incubated for 24 h at 38.5 °C, 5% CO_2_, and maximal humidity.

### 2.4. Determination of Nuclear Maturation Stage

**Figure 1:**
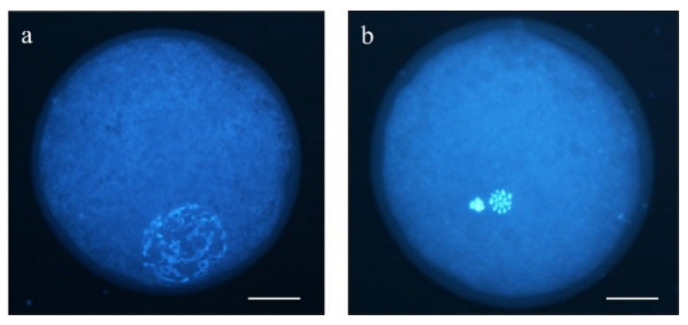
Representative images of (**a**) germinal vesicle (GV) stage oocyte and (**b**) MII oocyte. Scale bar = 20 µm.

Upon maturation, a total of 371 oocytes (5 replicates) were washed in PBS-PVA (#P4417 and #P8136), denuded from cumulus cells by gentle pipetting, and placed in a glass slide with a 1 µL drop of Slowfade™ (Invitrogen©, Thermo Fisher Scientific, Barcelona, Spain; #S36963) and 5 µg/mL of Hoechst 33342 (#14533) under a coverslip. After 10 min at room temperature, chromatin configurations were analyzed by fluorescence microscopy (Eclipse 80i, Nikon Instruments Europe, Amsterdam, The Netherlands). Oocytes showing a germinal vesicle (GV) chromatin configuration were considered immature, and those showing a metaphase plate and a polar body were categorized as matured metaphase II (MII) oocytes (Figure 1). 

### 2.5. Evaluation of Quality Parameters in Oocytes and Cumulus Cells

#### 2.5.1. Early Apoptosis Assessment

**Figure 2:**
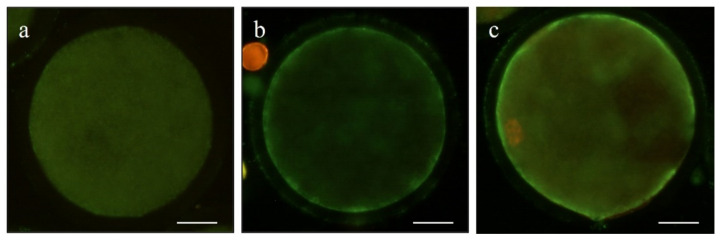
Representative images of (**a**) a viable sheep oocyte, (**b**) early apoptotic, and (**c**) dead. Scale bar = 20 µm.

Early apoptosis was evaluated in 108 mature oocytes (5 replicates) using Annexin V staining (Invitrogen©, Thermo Fisher Scientific, Barcelona, Spain; #A13199), following the manufacturers’ protocol. Denuded oocytes were incubated in Annexin V and 100 µg/mL of propidium iodide (PI; #P4864) for 20 min at 37 °C on a heated plate in the dark. After washing three times in PBS-PVA, oocytes were mounted on slides in a 1 µL drop of Slowfade™ and 5 µg/mL of Hoechst 33342. Oocyte nuclei were observed with an epifluorescence microscope (Eclipse 80i, Nikon Instruments Europe, Amsterdam, The Netherlands). Oocyte status was classified into the following categories: viable (Annexin V−/PI−), early apoptotic (Annexin V+/PI−), and dead (Annexin V−/PI+ and Annexin V+/PI+; Figure 2).

#### 2.5.2. Measurement of Reactive Oxygen Species (ROS) and Reduced Glutathione (GSH)

**Figure 3:**
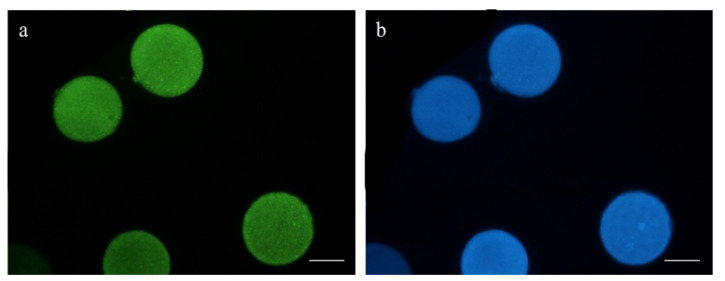
Representative images of intracellular (**a**) ROS and (**b**) GSH levels in mature oocytes. Scale bar = 50 µm.

A total of 110 mature oocytes (5 replicates) were incubated in 10 µM of CM-H_2_DCFDA (Thermo Fisher Scientific, Barcelona, Spain; #C6827) and 10 µM of Cell Tracker Blue (Thermo Fisher Scientific, Barcelona, Spain; #C12881) for 30 min at 38.5 °C in the dark and 5% CO_2_ to detect intracellular reactive oxygen species (ROS) and reduced glutathione (GSH), respectively. The oocytes were then washed thrice in PBS-PVA and placed on slides for evaluation. Fluorescence intensity was observed by epifluorescence microscopy (Eclipse 80i, Nikon Instruments Europe, Amsterdam, The Netherlands; Figure 3) and further evaluated using ImageJ 1.45s software (National Institutes of Health, Bethesda, MA, USA).

#### 2.5.3. Evaluation of Mitochondrial and Cortical Granule Distribution

To determine mitochondrial distribution patterns, 102 mature oocytes (5 replicates) were subjected to a double staining with MitoTracker© Red CMXRos (Thermo Fisher, Barcelona, Spain; #M7512), a mitochondrial-specific probe, and Hoechst 33342 to stain the chromosomes. Following IVM, oocytes were incubated for 30 min in PBS-PVA plus 500 nM of MitoTracker© Red CMXRos at 38.5 °C and 5% CO_2_ in the dark. The oocytes were then washed thrice under agitation for 5 min and mounted on slides with a 1 µL drop of Slowfade™ and 5 µg/mL of Hoechst 33342. Fluorescent images were recorded on a Nikon A1 confocal microscope (Nikon Instruments Europe, Amsterdam, The Netherlands). Lasers of 408 and 561 nm were used to excite Hoechst and MitoTracker Red dyes, sequentially. 

**Figure 4:**
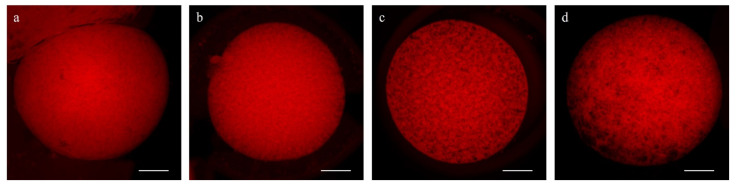
Representative images of mitochondrial distribution patterns at MII stage in IVM sheep oocytes. (**a**) Fine, (**b**) small, and (**c**) large granulations, and (**d**) clustered distribution. Scale bar = 20 µm.

Mitochondrial distribution patterns were classified as previously reported, with some modifications [24]: (1) fine pattern: with fine and small granulations spread homogeneously throughout the cytoplasm, and (2) granular pattern: with large granulations dispersed throughout the cytoplasm or heterogeneously distributed in clusters (Figure 4).

**Figure 5:**
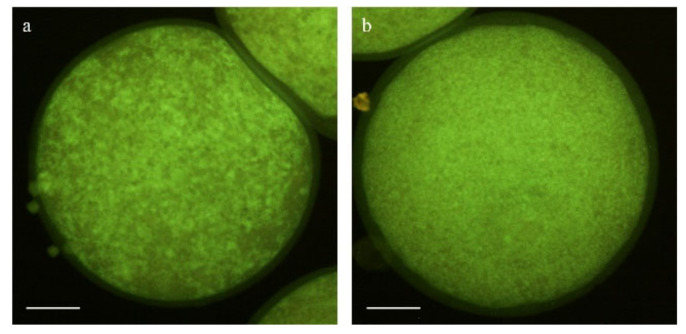
Representative images of cortical granules’ distribution patterns at the MII stage in IVM sheep oocytes. (**a**) Heterogeneous aggregated or clustered distribution, and (**b**) homogeneous fine distribution. Scale bar = 20 µm.

To identify the distribution pattern of cortical granules, a total of 79 denuded mature oocytes (4 replicates) were incubated in 4% paraformaldehyde (#P6148) for 1 h at room temperature. After washing thrice under a 5 min agitation in PBS-PVA, oocytes were permeabilized in 1% Triton (#T8787) for 1 h at room temperature and stained in 30 µL drops of 100 µg/mL of fluorescein isothiocyanate-conjugated peanut agglutinin (FITC-PNA; Invitrogen©, Thermo Fisher Scientific, Barcelona, Spain; #L21409) for 30 min at 37 °C on a heated plate and in the dark. After staining, oocytes were washed thrice in PBS-PVA, mounted on slides with 1 µL drops of Slowfade™ and 5 µg/mL of Hoechst 33342, and evaluated using a Nikon A1 confocal microscope (Nikon Instruments Europe, Amsterdam, The Netherlands). Lasers of 408 and 488 nm were used to excite Hoechst and FITC-PNA dyes, sequentially. Matured (MII) oocytes were classified into two groups according to the observed distributional pattern of the cortical granules, as previously described [25], with some modifications: Pattern I: aggregates or clusters of cortical granules distributed heterogeneously over the whole cytoplasm, and Pattern II: fine cortical granules distributed uniformly on the whole cytoplasm without the presence of aggregates (Figure 5).

#### 2.5.4. DNA Fragmentation Assay

**Figure 6:**
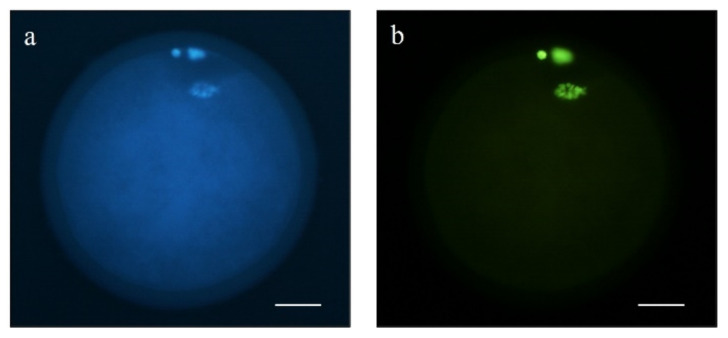
Representative images of a TUNEL-positive MII oocyte stained with (**a**) Hoechst 33342 and (**b**) fluorescein. Scale bar = 20 µm.

Terminal deoxynucleotidyl transferase-mediated dUTP nick-end labeling (TUNEL) was used to detect DNA fragmentation in mature sheep oocytes (4 replicates). A total of 75 oocytes fixed in 4% paraformaldehyde were permeabilized in 0.5% Triton X-100 for 1 h at room temperature. Then, the In Situ Cell Death Detection kit (#11684795910) was used to detect DNA strand breaks following the manufacturers’ instructions. Briefly, oocytes were placed in 30 µL drops of TUNEL reagent with deoxyuridine 5-trisphosphate (dUTP)-conjugated isothiocyanate fluorescein and incubated for 1 h at 37 °C. The positive control was pre-incubated with DNAse (0.2 U/µL; Thermo Fisher Scientific, Barcelona, Spain; #EN0521) for 1 h at 37 °C, while the negative control was incubated in the absence of deoxynucleotidyl transferase enzyme. After that, oocytes were washed thrice in PBS-PVA and placed on slides in a 1 µL drop of Slowfade™ with 5 µg/mL of Hoechst 33342. Samples were evaluated by epifluorescence microscopy (Eclipse 80i, Nikon Instruments Europe, Amsterdam, The Netherlands). Oocytes with DNA damage, e.g., with a fragmented nucleus, were classified as TUNEL-positive, and those without damage as TUNEL-negative (Figure 6).

#### 2.5.5. mRNA Transcript Analysis

The relative quantification of mRNA transcript abundances was measured by quantitative real-time PCR, as previously reported by Sánchez-Ajofrín et al., with minor modifications [26]. The RNA from 85 sheep oocytes was extracted using the Dynabeads^®^ Kit (Invitrogen, Waltham, CA, USA; #61012) in pools of approximately 10 oocytes and following the protocol by Bermejo-Álvarez et al. [27] (4 replicates). After the elution step, mRNA samples were reverse transcribed using the Fermentas™ First-Strand cDNA Synthesis Kit (Thermo Scientific, Barcelona, Spain; #K1612), as previously described [28]. After cDNA synthesis, the relative abundance of mRNA transcripts was examined by qPCR on a LightCycler 480 II instrument (Roche, Barcelona, Spain). A final volume of 20 µL was reached by adding 10 µL of NZYSpeedy qPCR Green Master Mix (Nzytech, Lisbon, Portugal; #MB22402), 400 nM each of forward and reverse primers (Supplementary Table S1), 2 µL of cDNA template, and 6.4 µL of nuclease-free water. The PCR amplification protocol consisted of 95 °C for 2 min, followed by 40 cycles of 95 °C for 5 s and 60 °C for 20 s. Samples were analyzed in duplicate, and reactions without any cDNA template were used as negative controls. Immediately after, a melting curve analysis was performed, and cycle threshold (Ct) values were recorded. The comparative Ct and 2 ^−ΔΔCT^ methods [29,30] were used to calculate the corresponding relative abundances of transcripts of interest: bone morphogenetic protein 15 (*BMP15*), DNA polymerase gamma 2, accessory subunit (*POLG2*), gap junction alpha-1 protein (*GJA1*, also known as *Cx43*), growth differentiation factor 9 (*GDF9*), nuclear respiratory factor 1 (*NRF1*), and transcription factor A, mitochondrial (*TFAM*). Quantification was normalized against that of the endogenous control (Peptidylprolyl Isomerase A (*PPIA*)).

#### 2.5.6. Cumulus Cells’ Analysis

Cumulus cells were collected from mature COCs and examined using a FlowSight^®^ Imaging Flow Cytometer (Amnis, Merck-Millipore, Germany), as previously described [28] (4 replicates). Briefly, samples were stained with 10 µM of YO-PRO-1 (Thermo Fisher Scientific, Barcelona, Spain; #Y3603) and 0.5 µM of PI to study viability, apoptosis, and mortality. Viable cells were recorded as YO-PRO-1−/PI−, while YO-PRO-1+/PI− were deemed apoptotic. Cells stained with PI were considered dead. For mitochondrial activity, cells were incubated with 200 mM of MitoTracker™ Deep Red (Thermo Fisher Scientific, Barcelona, Spain; #M22426) for 20 min at 38.5 °C in the dark and then stained with 10 µM of YO-PRO-1 and 0.5 µM of PI. Viable cells with active mitochondria were considered as MitoTracker+/YO-PRO-1-. To study GSH and ROS intracellular levels in viable cells, samples were incubated with 10 µM of Cell Tracker™ Blue (Thermo Fisher Scientific, Barcelona, Spain) and 10 µM of CM-H_2_DCFDA (Thermo Fisher Scientific, Barcelona, Spain) for 30 min at 38.5 °C, followed by 0.5 µM of PI staining. A compensation overlap was performed before each experiment, and 1000 events were acquired per sample. The raw data were analyzed using IDEAS^®^ software (AMNIS), and out-of-focus cells, debris, and cell clumps were excluded from the analysis (Supplementary Figure S1).

### 2.6. In Vitro Embryo Production

After IVM, control and Pre-IMV groups of 40–45 mature oocytes (*n* = 672, 5 replicates) were placed in 4-well dishes containing 500 µL of fertilization medium: synthetic oviductal fluid (SOF [31]), supplemented with 10% estrous sheep serum (ESS). Oocytes were subjected to in vitro fertilization (IVF) using frozen semen of one ram from the germplasm bank of the “Biology of Reproduction Group” of the Universidad de Castilla-La Mancha (UCLM), which is authorized for the collection and storage of sheep semen (ES07RS02OC). Thawed spermatozoa were separated using Percoll© (#P1644) density gradient (45%/90%) and capacitated for 15 min at 38.5 °C and 5% CO_2_ in fertilization medium. Then, spermatozoa (10^6^/mL) and oocytes were co-incubated at 38.5 °C in 5% CO_2_ and maximal humidity. After approximately 18 h post-insemination (hpi), putative zygotes were washed by repeated pipetting and transferred to 25 µL drops (about one embryo per µL) of culture medium (SOF supplemented with 3 mg/mL of bovine serum albumin (#A9647)), covered with mineral oil, and cultured until day 8 post-insemination (dpi) at 38.5 °C in a humidified atmosphere and 5% CO_2_, 5% O_2_, and 90% N_2_ in air. Cleavage and blastocyst rates were checked at 48 hpi and 6, 7, and 8 dpi, respectively.

### 2.7. Statistical Analysis

Statistical analyses were performed using the IBM SPSS 24.0 (IBM Corp.; Armonk, NY, USA) software. Data were tested for normal distribution (Kolmogorov–Smirnov and Shapiro–Wilk tests) and homogeneity of variances (Levene test). Data expressed as percentages were transformed into arcsine (or logarithm) for statistical analysis but reported as percentages to make them understandable. Concentration of cAMP, maturation rates, oocyte viability, apoptosis, mortality, oxidative status, mitochondrial distribution, embryo development (5 replicates), and mRNA transcript abundance, DNA fragmentation, cortical granules’ distribution, and cumulus cells’ activity (4 replicates) were analyzed by factorial ANOVA followed by the Bonferroni post hoc test. Differences with probabilities of *p* ≤ 0.05 were considered significant, and results are presented as mean ± SEM.

## 3. Results

### 3.1. Changes in Intra-Oocyte cAMP Levels after Incubation with Forskolin and IBMX during Pre-IVM

**Figure 7:**
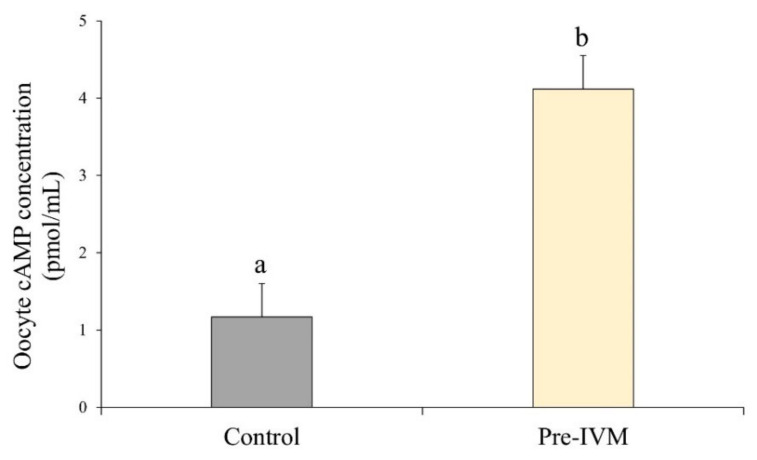
Effect of forskolin and IBMX during pre-IVM on intra-oocyte cAMP levels (pmol/mL). Results are expressed as mean ± SEM. ^a,b^ Different letters indicate differences between treatments.

After oocyte collection, culture of COCs for 2 h with forskolin and IBMX (pre-IVM treatment) resulted in higher (*p* < 0.05) intra-oocyte cAMP levels (4.12 ± 0.43 pmol/mL) compared to the control treatment (1.17 ± 0.43 pmol/mL; Figure 7).

### 3.2. Oocyte DNA Fragmentation Levels Are Affected by cAMP Modulators

**Figure 8:**
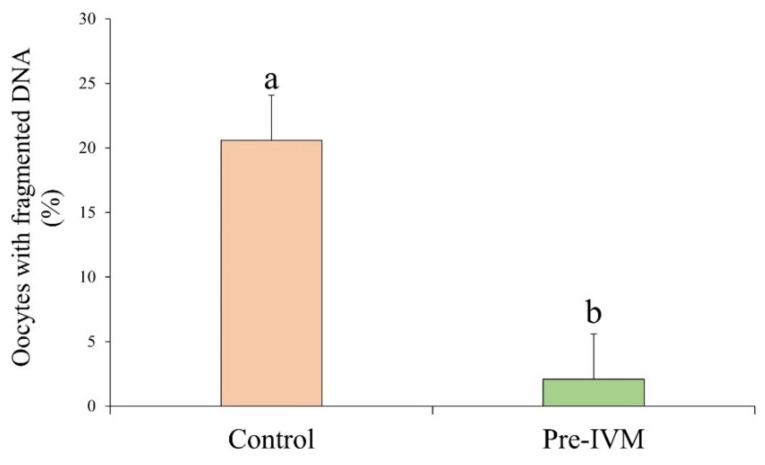
The effect of pre-IVM on the DNA fragmentation of matured sheep oocytes. Results are expressed as mean ± SEM. ^a,b^ Different letters indicate differences between treatments.

After IVM, the number of oocytes with fragmented DNA as measured by the TUNEL assay was significantly lower (*p* < 0.05) in pre-IVM (2.08% ± 3.51%) oocytes when compared to the control group (20.58% ± 3.51%; Figure 8).

### 3.3. Effect of Pre-IVM on Oocyte Live/Death, Apoptosis and Oxidative Status, and Relative Abundance of mRNA Transcripts after IVM

**Figure 9:**
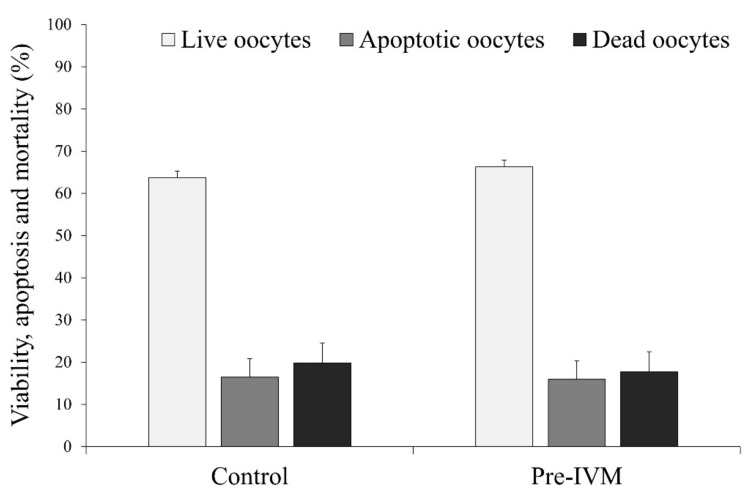
The effect of pre-IVM on the number of viable, early apoptotic, and dead sheep oocytes after IVM. Results are expressed as mean ± SEM.

The results did not find differences (*p* > 0.05) in the percentage of live, early apoptotic, and dead oocytes between the control (live = 63.76% ± 1.57%, apoptotic = 16.45% ± 4.38%, and dead = 19.79% ± 4.76%) and pre-IVM (live = 66.29% ± 1.57%, apoptotic = 15.96% ± 4.38%, and dead = 17.74% ± 4.76%; Figure 9). 

**Figure 10:**
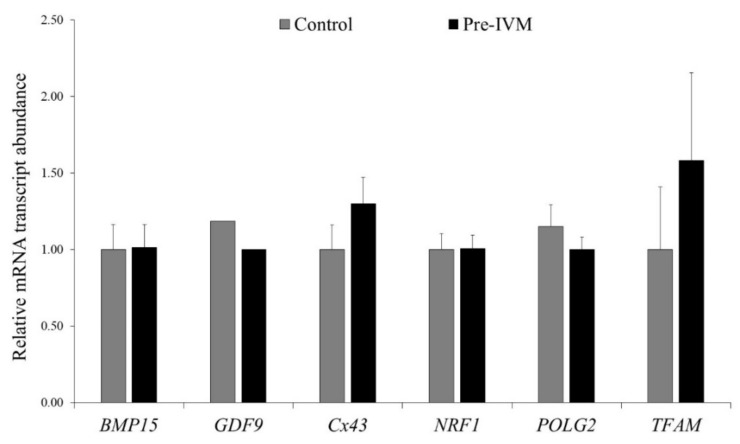
The effect of pre-IVM on the intracellular levels of ROS and GSH in matured sheep oocytes. Results are expressed as mean ± SEM.

The redox state of oocytes from both experimental groups was also similar (*p* > 0.05). Although not significant (*p* > 0.05), the intracellular ROS and GSH contents were lower and higher, respectively, in pre-IVM oocytes (ROS = 18.20 ± 16.95 and GSH = 82.23 ± 17.99) compared to the control (ROS = 44.78 ± 16.95 and GSH = 63.89 ± 17.99; Figure 10).

**Figure 11:**
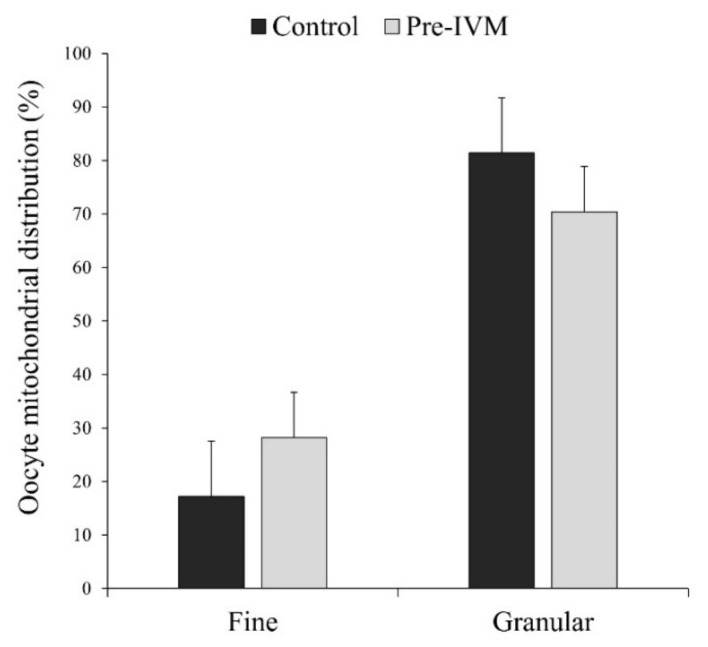
Relative mRNA transcript abundance pattern of genes of interest in sheep IVM oocytes subjected to pre-IVM. Results are expressed as mean ± SEM.

Cumulus cell-free oocytes were subjected to qPCR analysis to study mRNA transcripts related to meiotic competence and oocyte quality. As shown in Figure 11, no significant changes (*p* > 0.05) were detected for *BMP15*, *GDF9*, *Cx43*, *NRF1*, *POLG2*, and *TFAM* relative abundances between control and pre-IVM oocytes.

### 3.4. Effect of Pre-IVM on Cytoplasmic Maturation Features: Distribution of Organelles

**Figure 12:**
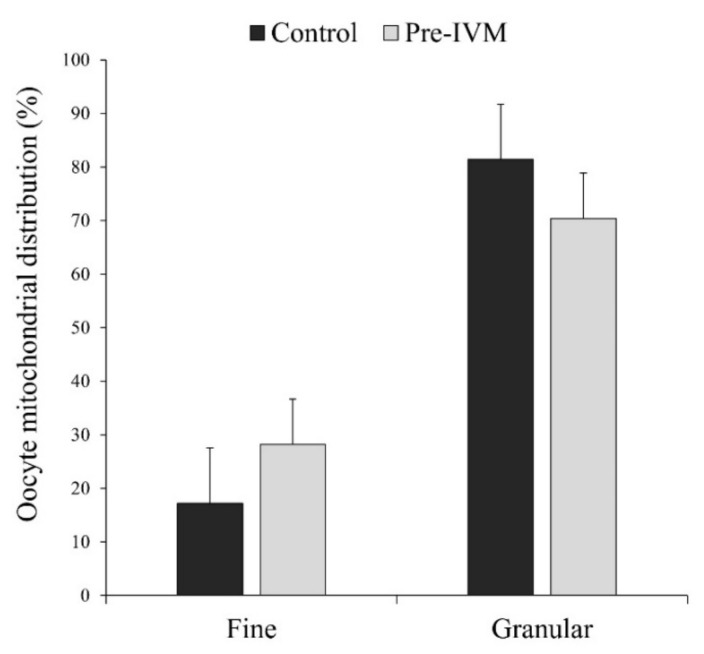
The effect of pre-IVM on the mitochondrial distribution patterns of matured sheep oocytes. Results are expressed as mean ± SEM.

In most MII oocytes, irrespective of the treatment, the distribution of mitochondria was homogenous granular or heterogeneous clustered (granular pattern; Figure 12), which has been considered a sign of maturation. More specifically, the granular pattern was observed in 81.40% ± 10.36% of the control oocytes and 70.38% ± 8.46% of pre-IVM oocytes, while the fine pattern (homogeneous fine) in 17.20% ± 10.36% of control oocytes and 28.23% ± 8.47% of pre-IVM (Figure 12). Despite this observation, oocytes from both experimental groups did not show a different mitochondrial distribution (*p* > 0.05) with respect to each other.

**Figure 13:**
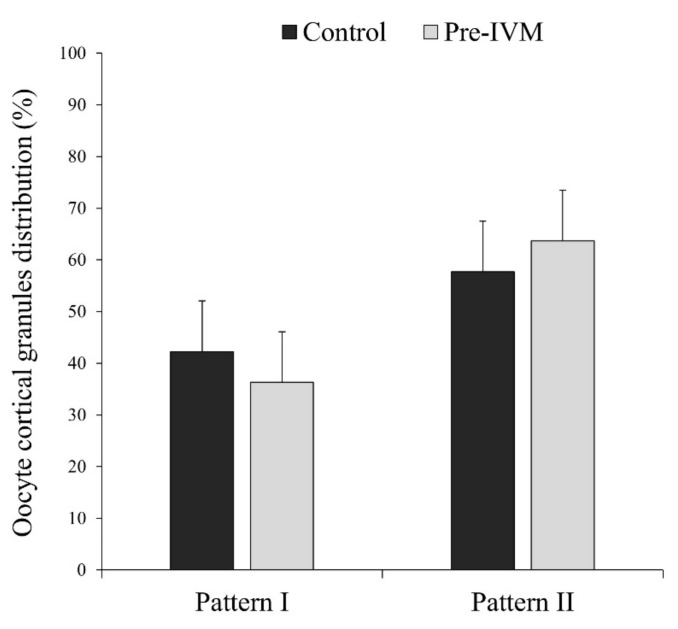
The effect of pre-IVM on the cortical granule distribution patterns of matured sheep oocytes. Results are expressed as mean ± SEM.

The percentage of cortical granule distribution patterns in MII oocytes is presented in Figure 13. Most of the mature oocytes showed pattern II (distributed uniformly over the entire cytoplasm without aggregates: control = 57.72% ± 9.79% and pre-IVM = 63.69% ± 9.79%). The rest of the MII oocytes (control = 42.27% ± 9.79% and pre-IVM = 36.31% ± 9.79%) presented pattern I (aggregates or clusters over the entire cytoplasm), which has been considered a sign of lack of maturation. However, the distribution of cortical granule migration patterns was similar (*p* > 0.05) in both experimental groups.

### 3.5. Effect of Pre-IVM on Subsequent Maturation and Developmental Competence of Sheep Oocytes

To determine the developmental consequences of forskolin and IBMX supplementation, following pre-IVM, COCs were subjected to standard IVM, IVF, and embryo culture to Day 8.

Although pre-IVM and control oocytes did not show differences (*p* > 0.05) in terms of oocyte maturation and cleavage rates, the percentage of blastocysts from both the initial number of oocytes and cleaved embryos at 48 hpi was significantly increased (*p* < 0.05) in the pre-IVM group (Table 1).

### 3.6. Cumulus Cells’ Quality Parameters from In Vitro Matured COCs with and without Pre-IVM Culture

As shown in Table 2, we assessed cumulus cells’ live/death status, apoptosis, number of active mitochondria, and intracellular ROS and GSH content via flow cytometry. In all parameters, pre-IVM treatment showed similar values (*p* > 0.05) to the control treatment.

## 4. Discussion

To the best of our knowledge, this is the first study to examine the effect of cAMP modulators on a series of nuclear and cytoplasmic maturation features of IVM sheep oocytes, including the redistribution of cytoplasmic organelles, DNA fragmentation, transcription of mRNAs, and cumulus cells’ activity. For this, immature sheep oocytes were cultured for 2 h with IBMX, a PDE inhibitor that halts cAMP degradation, and forskolin, a potent adenylate cyclase activator. Following IVM, oocytes were fertilized and cultured in vitro up to the blastocyst stage.

Considering that cAMP modulators aim to synchronize nuclear and cytoplasmic maturation events [32], we focused on the underlying cellular mechanisms through which forskolin and IBMX could influence oocyte quality and developmental competence in sheep. Surprisingly, important parameters such as mitochondrial and cortical granule distribution did not show differences between pre-IVM and control groups. In fact, most oocytes exhibited the typical pattern of an oocyte at the MII stage. Notwithstanding these initial data, we did observe a remarkable reduction of DNA fragmentation in pre-IVM oocytes. This observation provided a vital clue on the involvement of DNA integrity in the cAMP modulators’ ability to influence oocyte development in vitro. 

During germinal vesicle breakdown (GVBD), physiological DNA single- and double-strand breaks (SSBs and DSBs, respectively) form during the displacement and separation of chromosomes. These damaged DNA fractures need to be repaired quickly to ensure the successful completion of maturation and preserve genomic stability in the oocyte [33]. However, due to the nature of in vitro culture conditions, oocytes are highly vulnerable to the accumulation of environmental damage, including DNA damage [34]. Reaching the threshold at which DNA damage cannot be repaired rapidly and effectively would negatively affect the oocyte and lead to genetic abnormalities and defective embryo development [33,35]. 

In our study, control oocytes reached the MII stage at the end of the culture period, similar to pre-IVM oocytes. These results are in agreement with recent studies in cattle [36] and horse [37], and were not surprising since Marangos et al., recently showed that the presence of DSBs at the G2 phase of meiosis did not prevent mouse oocytes from entering metaphase I unless the damage was particularly severe [38]. It is therefore feasible that the amount of the initial DNA damage was similar between pre-IVM and control oocytes; however, forskolin and IMBX acted more efficiently in removing this damage via their marked effect on cAMP modulation. Besides, maintaining meiotic arrest during pre-IVM may eventually give oocytes more time to repair the DNA damage. Previously, several studies have reported a link between the cAMP signaling pathway and increased DNA repair in melanocytes [39,40,41]. Furthermore, Passeron et al., demonstrated that the use of forskolin in human skin significantly reduced DNA damage after 6, 24, and 48 h of exposure [42]. We, therefore, hypothesized that artificially increasing oocyte cAMP levels before IVM might regulate subsequent DNA repair mechanisms and eventually enhance DNA integrity in the oocyte at the MII stage. In this context, Sun et al., revealed that inducing DSBs in mouse COCs prevents the meiotic resumption of oocytes by promoting cumulus cell cAMP synthesis, hereby showing a relationship between DSBs and increased cAMP synthesis [43]. However, it is still unknown what molecular mechanisms are involved in the association between cAMP modulation and DNA repair capacity in the oocyte, and it requires further research.

With reduced DNA fragmentation, pre-IVM oocytes developed to the blastocyst stage to a greater extent than control oocytes. Previous studies suggested that oocytes may accumulate sufficient factors, such as mRNAs and proteins, to preserve the genome until the zygotic genome is activated [44,45]. These factors may also prevent the accumulation of mutations in DNA repair genes compromising blastocyst development and increasing blastomere apoptosis [34]. Thus, forskolin and IBMX may help to provide a series of factors that ultimately enhance the embryos’ preimplantation development. Besides, previous studies in other species showed that forskolin and IBMXs’ beneficial effects on embryo development are also related to an increased ATP, and oxygen consumption [22,46], modulation of lipid content [19], and reduced spindle abnormalities [22], amongst others. Along with DNA integrity, these parameters have often been regarded as good indicators of oocyte quality [47,48,49].

## 5. Conclusions

Based on this evidence, the results suggest that the impact of intra-oocyte cAMP modulation on subsequent in vitro embryo development is linked to a decrease in oocyte DNA damage after IVM. This previously unappreciated action of the cAMP modulators forskolin and IBMX may reflect an integral role in ensuring the maternal genomes’ integrity required for the correct initiation of embryonic transcription in vitro. Therefore, ascertaining whether gamete exposure to cAMP modulators before IVM induces specific DNA repair or cell cycle checkpoint genes could help to develop improved methods for assisted reproduction technologies in animal and clinical research. Nevertheless, since post-mortem ovaries were used in the present study, further investigation will be necessary to account for the variability of the oocyte source.

## Figures and Tables

**Table 1:** The effect of pre-IVM on oocyte meiotic and developmental competence in sheep.

Treatment	Germinal Vesicle (%)	Maturation MII (%)	Cleaved Embryo at 48 hpi (%)	Expanded Blastocyst (%)	
				Total	Cleaved
Control	6.50 ± 2.26	87.85 ± 1.52	55.28 ± 4.00	18.22 ± 1.47 ^a^	32.88 ± 1.73 ^a^
Pre-IVM	3.43 ± 2.26	91.75 ± 1.52	55.01 ± 4.00	23.89 ± 1.47 ^b^	45.16 ± 1.73 ^b^

Data are expressed as mean ± SEM. ^a,b^ Different letters indicate differences (*p* ≤ 0.05) between treatments.

**Table 2:** The impact of pre-IVM on cumulus cells in sheep.

Treatment	Viable Cells (%)	Apoptotic Cells (%)	Dead Cells (%)	Active Mitochondria (%)	ROS Levels (Fluorescence Intensity)	GSH Levels (Fluorescence Intensity)
Control	77.7 ± 2.4	5.8 ± 1.0	16.1 ± 1.7	61.2 ± 1.2	6906.5 ± 339.1	8915.7 ± 738.7
Pre-IVM	80.6 ± 2.4	7.60 ± 1.0	11.2 ± 1.7	64.2 ± 1.2	6950.0 ± 339.1	9022.1 ± 738.7

Data are expressed as mean ± SEM.

## Supplementary Materials

The following are available online at https://www.mdpi.com/article/10.3390/ani11092512/s1, Table S1: Details of primers used for qPCR. Figure S1: Representative images of cumulus cells’ populations regarding (A) viability, apoptosis, and mortality, (B) active mitochondria, and (C) intracellular levels of ROS and GSH using flow cytometry.
