# Supplementary material for: cAMP Modulators before In Vitro Maturation Decrease DNA Damage and Boost Developmental Potential of Sheep Oocytes
Source: Animals (Basel). 2021 Aug 26;11(9):2512. doi: 10.3390/ani11092512 (PMC8467748; doi:10.3390/ani11092512)
Supplement: Supplementary file 1 [file animals-11-02512-s001.zip › animals-1295527-supplementary.pdf]

animals

Supplementary material

Table S1. Details of primers used for qPCR.

Gene	Gene Function	Primer Sequence (5′–3′)	Product Size (bp)	Accession No.
PPIA	Reference gene	F- TCAACCCCACCGTGTTCTTC<br>R- GTCACCACCCTGGCACATAA	194	NM_001308578.1
BMP15	Oocyte maturation	F-CTACGACTCCGCTTCGTGTGT<br>R-AGTGCCATGCCACCAGAAC	69	NM_001031752.1
GDF9		F-GAAGTGGGACAACTGGATTGTG<br>R-CCCTGGGACAGTCCCCTTTA	71	NM_174681.2
Cx43	GAP junctions	F-TGCCTTTCGTTGTAACACTCA<br>R-AGAACACATGAGCCAGGTACA	143	NM_174068.2
NRF1	Transcription factor	F-CTGTCGCCCAAGTGAATTATTCG<br>R-TGTAACGTGGCCCAGTTTTGT	67	NM_001098002.2
POLG2		F-CTTCTGGGAAACTACGGGAGAAC<br>R-GTAGCCTCTTGTTTACCAGATCCA	84	NM_001075191.1
TFAM		F-CACTGGGAAAGTCAGGAGCG<br>R-ACATACGCAAAACTAAAGGGGG	76	XM_027962472.1

F, forward primer; R, reverse primer.

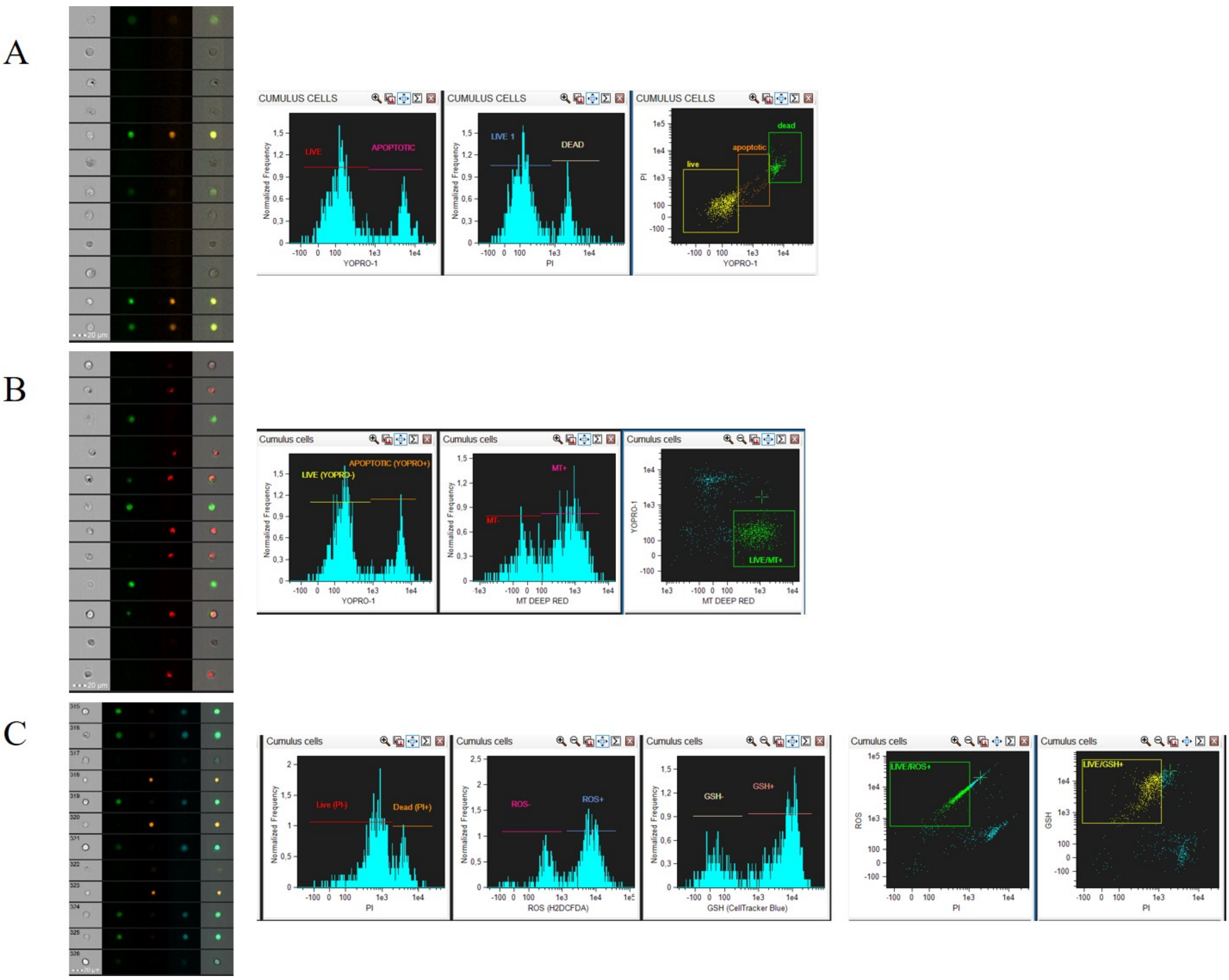

Figure S1. Representative images of cumulus cells populations regarding (A) viability, apoptosis and mortality, (B) active mitochondria, and (C) intracellular levels of ROS and GSH using flow cytometry.
